# Enhancing Starch Film Properties Using Bacterial Nanocellulose-Stabilized Pickering Emulsions

**DOI:** 10.3390/polym16233346

**Published:** 2024-11-29

**Authors:** Natália Tavares de Almeida, André Luís Sousa Pereira, Matheus de Oliveira Barros, Adriano Lincoln Albuquerque Mattos, Morsyleide de Freitas Rosa

**Affiliations:** 1 Department of Food Engineering, Federal University of Ceará (UFC), Fortaleza 60455-760, CE, Brazil; nataliatavares1998@hotmail.com; 2 Department of Organic and Inorganic Chemistry, Federal University of Ceará (UFC), Block 940, Fortaleza 60455-760, CE, Brazil; andre110487@gmail.com; 3 Department of Chemical Engineering, Federal University of Ceará (UFC), Block 709, Fortaleza 60455-760, CE, Brazil; matheus_barros@outlook.com; 4 Embrapa Tropical Agroindustry, Rua Dra. Sara Mesquita 2270, Fortaleza 60511-110, CE, Brazil; adriano.mattos@embrapa.br

**Keywords:** biopolymers, Pickering emulsions, corn starch, packaging

## Abstract

This study aimed to address issues related to hydrophilicity, barrier properties, and mechanical performance in starch-based films by incorporating Pickering emulsions stabilized with nano-fibrillated bacterial cellulose (BC). Emulsions were added to the film-forming suspension at varying concentrations (1.0%, 2.5%, 5.0%, and 7.5% *v*/*v*) for comparison. The films were evaluated using water vapor permeability (WVP), contact angle, Fourier Transform Infrared Spectroscopy (FTIR), and tensile tests. The results showed a significant reduction in film hydrophilicity, with the contact angle increasing from 49.7° ± 1.5 to 71.0° ± 1.4, and improved water vapor barrier properties, with WVP decreasing from 0.085 ± 0.04 to 0.016 ± 0.01 g·mm/h·m^2^·kPa. FTIR analysis confirmed the successful incorporation of the emulsion into the starch matrix. Among the tested concentrations, 2.5% provided an optimal balance, increasing hydrophobicity while maintaining mechanical strength. These findings demonstrate that Pickering emulsions are an effective strategy for enhancing the functional properties of starch films.

## 1. Introduction

With a growing trend of environmentally conscious consumers, the food sector—responsible for 50% of plastic packaging usage [1], including films and coatings—stands out as one of the most promising sectors for the incorporation of biobased materials like biopolymers into its production line.

While the use of biopolymers in film production presents several advantages, there are limitations. These include typically weak mechanical properties and high water vapor permeability, which are associated with the hygroscopic and hydrophilic nature of most biopolymers, as well as common plasticizers like glycerol and sorbitol [2]. The ability to block water vapor is crucial for many food packaging applications, as poor performance in this area can negatively affect consumer acceptance.

Such is the case with starch-based packaging materials, which have been explored as alternatives to plastics because starches are natural film-forming polymers that are abundant, affordable, safe, tasteless, biocompatible, and biodegradable [3]. However, their application is limited due to inherent deficiencies in starch, such as its hydrophilic nature and susceptibility to retrogradation [4], as well as issues arising from the film preparation process, including increased water vapor permeability and low mechanical resistance [5].

To address the deficiencies identified in starch films, several techniques have been applied. Cold plasma treatment is primarily superficial and alters mechanical properties but not barrier properties [6]. Similarly, ultraviolet light treatment induces the formation of cross-links on the film’s surface, improving mechanical properties and increasing hydrophobicity up to a certain frequency, as provided by a cross-link machine. Ultrasonic treatment enhances the homogenization of the film-forming matrix by breaking down very small starch particles and preventing the appearance of cracks. However, high energy (intensity, prolonged use) can degrade the starch chains, resulting in a brittle film [7]. The use of nanostructured additives, mainly cellulose nanocrystals, requires a chemical production process that incorporates functional groups to improve interaction with the starch film-forming matrix, necessitating multiple purification steps [8].

Previous studies have demonstrated the potential of bacterial cellulose (BC) as a valuable additive for film improvement. For instance, Liang et al. (2023) [9] developed composite films of starch and mutated bacterial cellulose that demonstrated good mechanical strength without compromising performance. Similarly, Abral et al. (2021) [10] studied composite films incorporating BC nanocellulose, starch, and chitosan for antimicrobial, as well as edible films suitable for food packaging. Additionally, Jiang et al. (2023) [11] investigated flexible, semi-transparent bacterial cellulose films resistant to water, vapor, oxygen, ultraviolet light, and foodborne pathogens.

Another common approach to enhancing the water vapor permeability (WVP) of films involves incorporating emulsified hydrophobic substances, such as waxes, oils, and essential oils, which typically rely on hydrophilic matrices. This approach also facilitates the integration of hydrophobic agents that have beneficial properties, such as vitamins, making it easier to introduce antioxidant and antimicrobial properties that would be difficult to achieve in purely hydrophilic matrices [12,13].

Studies have highlighted the potential of Pickering emulsions as effective systems for enhancing the water vapor barrier capabilities of bio-based films by affecting their hydrophilic/hydrophobic balance. Pickering emulsions differ from traditional emulsions in that they are stabilized by solid particles instead of surfactants (“surfactant-free”) [14]. As a result, they generate considerable interest because of their stability and because they do not contain compounds like some synthetic emulsifiers that could raise problems from environmental, pharmaceutical, and food safety perspectives.

However, the inclusion of Pickering emulsions tends to negatively impact the tensile strength of these films. Moreover, to effectively enhance the water vapor barrier properties, it is crucial that the oil droplets are uniformly distributed within the matrix. If the dispersion is uneven, it can lead to the formation of discontinuities, which might facilitate water vapor diffusion, resulting in a counterproductive outcome. Further research is necessary to develop Pickering-based structures that retain the benefits of hydrophobic compounds in films without compromising their mechanical properties and, ideally, even enhancing them [13].

To overcome these limitations, this study explores incorporating various concentrations of Oil-in-Water Pickering emulsions (O/W), stabilized by bacterial cellulose (BC), into starch films. BC, known for its amphiphilic nature [15], biodegradability, and biocompatibility, can be processed into nanofibrils (BC-NF) by reducing its fibrillar structure through physical methods. These nanocelluloses offer significant potential for stabilizing food emulsions due to their unique properties [16]. The objective is to investigate the effects of these emulsions on the technological properties of the starch films, with particular attention to barrier properties, hydrophilic character, and mechanical strength. By systematically analyzing these properties, this research seeks to provide valuable insights into optimizing starch-based films for potential applications in sustainable food packaging, ultimately contributing to the reduction in plastic waste and environmental impact.

## 2. Materials and Methods

### 2.1. Materials

The bacterial cellulose membranes were donated by the Seven Business Group to Embrapa Tropical Agroindustry. Corn starch (MAIZENA, Unilever Brazil, São Paulo, Brazil) and sunflower oil (LIZA, Cargill, Curitiba, Brazil), on the other hand, are of commercial origin. The TEMPO (2,2,6,6-tetramethylpiperidine-1-oxyl, 156.25 mol.g^−1^) radical and ethanol were obtained from Sigma Aldrich (São Paulo, Brazil). The NaOH, NaBr, and NaOCl were obtained from Dinâmica (Caxias do Sul, Brazil). The reagents were used without further purification.

### 2.2. Obtaining Colloidal Suspensions of Bacterial Cellulose Nanofibrils (BCNFs)

BCNFs were obtained through the methodology of Pereira et al. (2020) [17]. After the complete dissolution of NaBr and TEMPO, 1 g of BC (dry basis) was added. After homogenization, NaOCl (5.0 mmol) was added slowly, under stirring (1000 rpm) and at 25 ± 1 °C. The pH was adjusted to 10.0–10.5, and the reaction continued for 150 min. Ethanol (60 mL) was added to stop the reaction, and the characteristic yellow color of the reaction changed to white. The oxidized BC (BCOX) was washed with 200 mL of H_2_O, centrifuged (4 °C, 24,456× *g*, 15 min, 2×; Hitachi Koki Himac CR22GIII, Tokyo, Japan), and the formed hydrogel stored in a refrigerator (4 °C). The final concentration of the material was adjusted to 1% *m*/*v*, and the material was defibrillated in a colloidal mill (Meteor Rex Inox I-V-N) for 10 minutes. The formed nanofibrils (BCNF) were stored in a refrigerator (4 °C).

### 2.3. Preparation of Pickering Emulsions

For the Pickering O/W emulsion, a 10/90 ratio was adopted: 10% oil phase containing sunflower oil to 90% aqueous phase containing water and BCNF. The mixture was homogenized using a high-shear force mixer (Ultra Turrax T25 IKA, Staufen, Germany) at 10,000 rpm for 10 min.

### 2.4. Incorporation of Pickering Emulsions into Starch Films

The emulsion was incorporated in different concentrations in the starch films: 1.0% *v*/*v*, 2.5% *v*/*v*, 5.0% *v*/*v*, and 7.5% *v*/*v*. The films, in turn, were prepared according to the casting technique by Yang and Paulson (2000) [18], and the control composition was based on the literature by Donhowe and Fennema (1994) [19], maintaining a starch matrix at 5% *m*/*v*. Initially, water was heated to 85 °C, and under magnetic stirring, the starch was added slowly to avoid the appearance of lumps. After complete dissolution, the temperature was reduced to 60 °C, and glycerol and emulsion were added. At this same temperature, the film-forming solution was degassed in a rotary evaporator and then poured onto glass plates (30 cm × 60 cm) covered with mylar film and adjusted to a height of 1 mm using a millimeter bar. The composition of the films is described in Table 1.

### 2.5. Conductometric Titration and Zeta Potential of BCNFs

To confirm the presence of acidic groups in the cellulose chain, a conductimetric titration was performed [20,21]. The content of carboxylate was determined with volumes V0 and V1, the volume of NaOH required to neutralize the strong acid (HCl) and weak acid (–COOH), respectively, obtained from the curve conductivity measurement, based on Equation (1):

C = ((V_1_ − V_0_) × C_NaOH_)/m (1)

where C_NaOH_ is the concentration of NaOH used in the titration and m is the dry mass of the sample. A suspension of nanofibrils was diluted (1:50 *v*/*v*) with distilled water and then the Zeta potential was analyzed using 3000 Zetasizer Nano ZS (Malvern Instruments Ltd., Worcestershire, UK) at 25 °C.

### 2.6. Transmission Electronic Microscopy (TEM)

For TEM analysis, the BCNFs suspension was dispersed in Milli-Q water (1.25 g/L) and assembled on 300 mesh grids coated with formvar with the addition of a contrasting solution phosphotungstic acid (0.1% *w*/*v*). The grids were visualized under the microscope scanning electronics Tescan Vega 3 (Tescan, Brno, Czech Republic), with a STEM detector under an acceleration voltage of 30 kV. The average diameters of the nanofibrils were determined using the Gimp 2.6 software.

### 2.7. Average Particle Diameter in the Emulsion

The emulsion was observed using an optical microscope (ZEISS AXIO Imager A2, ZEISS, Oberkochen, Germany) equipped with a camera (ZEISS AxioCam ICc 5, ZEISS, Oberkochen, Germany) at two time points: immediately after preparation (day 0) and after a 15-day interval. A drop of the sample was placed onto a glass slide and carefully spread on a coverslip. The area of each droplet was measured using the ImageJ software (Version 1.54), and the mean diameter weighted by the particle area, referred to as D_32_, was calculated using Equation (2):

D_32_ = (Σn_i_d_i_^3^)/(Σn_i_d_i_^2^) (2)

where n_i_ is the number of detected particles with diameter d_i_.

### 2.8. Micrographs of Starch Films

The micrographs of the films were obtained by optical microscopy, using a Binocular Biological Microscope LED Nikon Eclipse E200 (Nikon, Tokyo, Japan). CFI E Plan Achromatic 4X objective lens (0.10/30). The micrographs of the emulsions were obtained using an optical microscope (ZEISS AXIO IMAGER A2, ZEISS, Oberkochen, Germany) equipped with a camera (ZEISS AxioCam Icc 5, ZEISS, Oberkochen, Germany).

### 2.9. Water Vapor Permeability (WVP)

The WVP determination was performed based on the E96-00 method (ASTM, 2000) [22]. Eight replicates were analyzed, and silica gel was used as a desiccant in an Arsec DCV040 vertical desiccator, in addition to water inside the permeation cells. All eight measurements were taken within 24 h, with an interval of at least 1 h between each weighing. The experiment was conducted at 25 °C and a relative humidity of 55%.

### 2.10. Water Contact Angle

Water contact angles were determined using an optical contact meter (GBX Instrumentation Specifique, GBX Instrumentation, Miami, FL, USA), in which a drop of Milli-Q water was deposited on the surface of each film. The 2 × 2 cm samples were fixed on glass support to capture the image by a Pixe Link Nikon camera (Nikon, Tokyo, Japan) at the moment the drop touched the surface, as well as measuring the angle. Measurements were performed in quadruplicate.

### 2.11. Fourier-Transform Infrared Spectroscopy (FTIR)

The surface spectrum of the starch films added with Pickering emulsion was performed in a Perkim-Elmer^®^ model Spectrum Two spectrometer in the attenuated total reflection (ATR) mode in the wavelength range: 4000–650 cm^−1^, 32 scans were made with a resolution of 4 cm^−1^.

The main peaks were normalized relative to the starch peak at 995 cm⁻^1^ using the following equation:

I_N_ = I_TP_/I_RP_, (3)

where I_N_ is the normalized intensity of the peak, I_TP_ is the original intensity of the targeted peak, and I_RP_ is the intensity of the reference peak (in this study, 995cm^−1^).

### 2.12. Tensile Tests of Starch Films

The films were cut into 11.9 mm wide × 10 cm long strips and were kept in a desiccator with Mg(NO_3_)_2_ at 23 °C and a relative humidity of 50% for 48 h. The assays were performed on a Universal Testing Machine DL10000 (EMIC, Instron, Paraná, Brazil) following ASTM D882-12 (2012) using a CCE500N load cell (EMIC, Instron, Paraná, Brazil) and a rate of grip separation of 50 mm/min. Measurements were performed in quintuplicate.

### 2.13. Statistical Analysis

The results were submitted to statistical analysis performed using the Tukey Test (*p* < 0.05) carried out in the STATISTIC 7.0 software, which allowed the comparison between the mean values obtained for each result.

## 3. Results and Discussion

### 3.1. Microscopic Analysis

**Figure 1:**
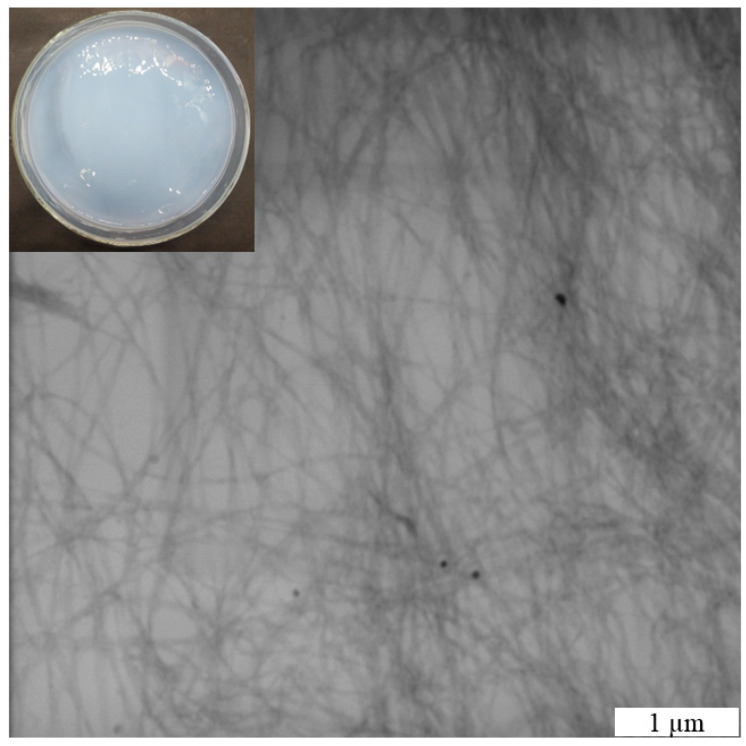
Micrograph of bacterial cellulose nanofibrils. Suspension in Petri dish (insert).

The microscopic images obtained via TEM showed that BCNFs were individually dispersed (Figure 1). Nanofibrils displayed an average diameter of 46.0 ± 14.8 nm and lengths spanning several micrometers. Oxidation resulted in the formation of carboxylate groups (1.13 mmol COO^−^.∙g^−1^ cellulose, equivalent to D.O. = 18.56%), which in turn induced electrostatic repulsions among the cellulose chains, thereby facilitating fibrillation and dispersion in aqueous media. Consequently, stable, viscous, opaque, and white gel dispersions of BCNFs were achieved. Nanofibrils exhibited a high (negative) surface charge (−60.2 mV), effectively mitigating instability phenomena attributed to the prevailing electrostatic repulsion forces.

**Figure 2:**
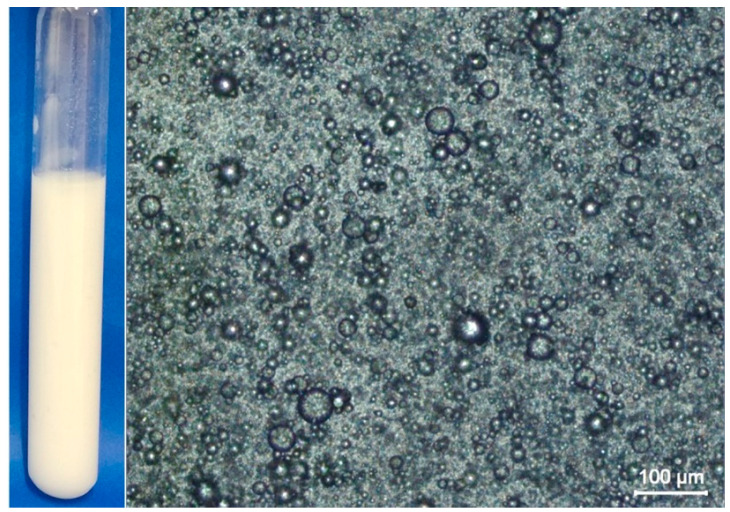
Optical microscopy of Pickering emulsion stabilized by BCNF after preparation at room temperature. The scale bar is 100 μm.

The optical microscopy analysis of the Pickering emulsion clearly showed the dispersion of oil droplets within the continuous phase (Figure 2). The oil droplets exhibit diverse sizes and arrangements relative to each other, showcasing a range of dimensions, including the merging of two or more droplets and the presence of isolated ones. On day 0, the oil droplets displayed a D32 value of 9.0 µm and 15.2 µm after 15 days. Although the D32 value increased, the overall stability of the emulsion was not significantly impacted, as the negative charge from the BCNF provided electrostatic stabilization to the colloidal system [23].

**Figure 3:**
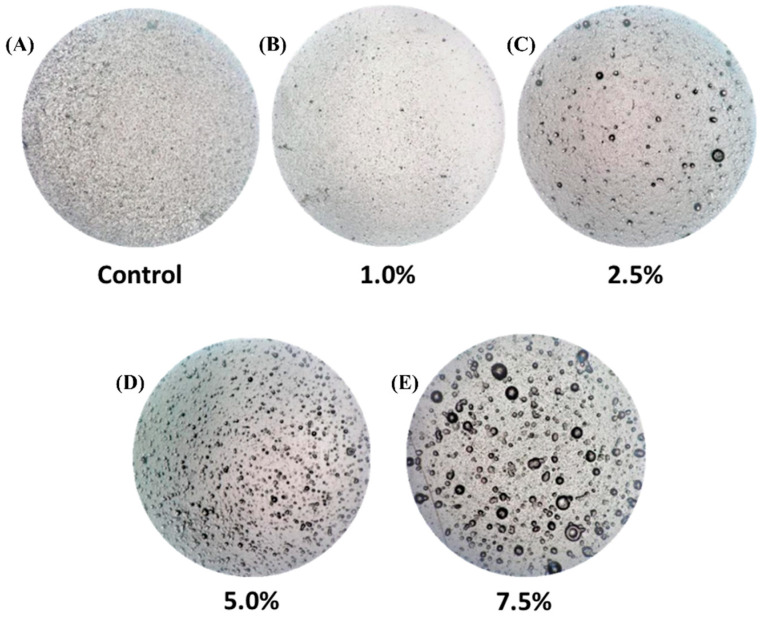
Micrographs of the films with different concentrations (1–7.5 % *v*/*v*) ((**A**–**E**), respectively) of emulsion added (4×/0.10 magnification).

Figure 3 shows the visual appearance of each film, where oil droplets are observed as quasi-spherical discontinuities. The size and distribution of these droplets vary among the formulations. In the 2.5% (*v*/*v*) film, larger droplets are present alongside smaller ones, resulting in a less uniform distribution. In the 7.5% (*v*/*v*) formulation, the droplets appear even larger and more irregularly distributed. Conversely, the 5.0% (*v*/*v*) Pickering emulsion film exhibits a more uniform visual appearance, characterized by droplets of similar size and a more orderly arrangement.

### 3.2. Fourier-Transform Infrared Spectroscopy (FTIR)

**Figure 4:**
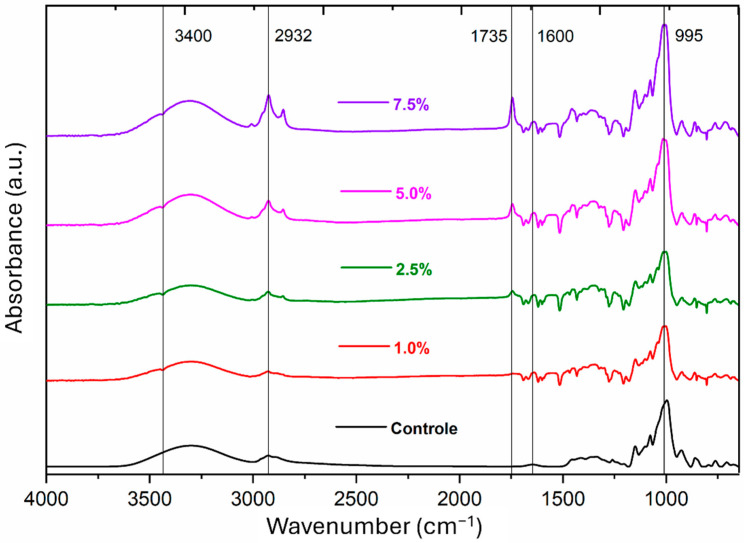
FTIR analysis of the control starch film and the starch films added to Pickering emulsion.

The FTIR analysis allowed observation, through the obtained spectra, of the presence of characteristic functional groups of the polymeric matrix, as well as of the added Pickering emulsion. Next, the main bands identified will be described (Figure 4).

Apparent in all spectra, the absorption bands close to 3400 cm^−1^ show the presence of hydroxyl groups, which can come from both cellulose and starch [24]. This explains why it appears both in the spectrum of the control film and in the others. The band at 2932 cm^−1^ refers to the CH functional group, indicating stretching vibration of the CH_3_ and CH_2_ groups, present both in starch [25] and in sunflower oil [26]. The normalized intensities of these peaks increase as the percentage of emulsion—and thus the oil content—rises (Table 2).

The region at 1735 cm^−1^ appears subtly from the 1.0% (*v*/*v*) formulation. The band is attributed to the C=O group of esters [27] present in sunflower oil. It gradually increases, forming an acute peak of absorption evidenced by the spectrum of 7.5% (*v*/*v*) and by the increasing values of the intensity of the normalized peaks (Table 2), which is coherent with the increase—also gradual—in the emulsion concentration, and therefore, the oil concentration. The band at 1600 cm^−1^ refers to the vibration of carboxylate groups [28] due to the oxidation carried out to obtain bacterial cellulose nanofibrils. Therefore, this band only appears in the spectra of starch films with added Pickering emulsion.

Finally, the region at 995 cm^−1^ is described as the amorphous part of the starch structure due to the amount of water that interacts with intermolecular hydrogen bonds [29]. The intensity of the band can vary according to the type of starch due to the different ways in which interactions occur due to the ratio of amylose/amylopectin present in each type of starch [30].

### 3.3. Water Vapor Permeability (WVP)

The addition of Pickering emulsion resulted in films exhibiting decreased water vapor permeability (WVP, Table 3), as expected due to the hydrophobic character of the oily phase, changing the hydrophobic–hydrophilic ratio of the films. Notably, formulation 5.0% showed the lowest WVP value (0.016 ± 0.01 g∙mm/h∙m^2^∙kPa). Interestingly, this value is statistically similar to that of the 1.0% (*v*/*v*) formulation, suggesting that even a small addition of Pickering emulsion (1.0% *v*/*v*) can significantly reduce WVP in starch films. Successful reduction in WVP in films relies on factors such as the dispersion of oil droplets in the matrix, which must be well executed to prevent phenomena like coalescence. Additionally, the nature of the oil used in the emulsion plays a crucial role, as WVP decreases with increasing chain length and degree of lipid saturation. Therefore, it can be inferred that the Pickering emulsions were effectively dispersed in the material, and the use of sunflower oil was suitable for this application [13].

### 3.4. Hydrophobicity

**Figure 5:**
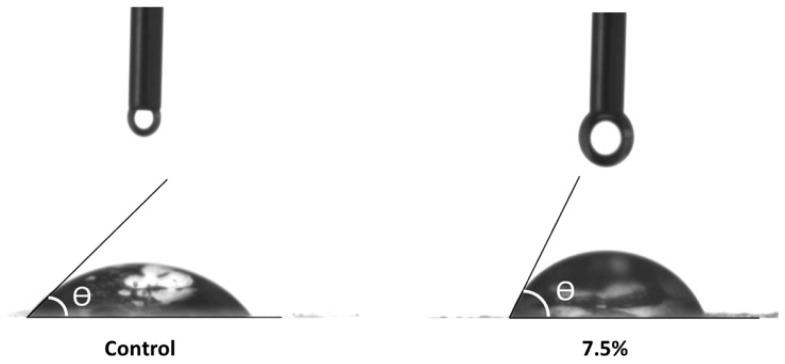
The moment when the drop touches the hydrophilic and hydrophobic surfaces.

The presence of emulsion significantly affected the surface hydrophobicity (Table 3) in concordance with previous reports of Pickering emulsions increasing water contact angle values of films [31]. From a statistical point of view, however, the tested formulations did not differ from each other. Thus, it can be attested that with only 1.0% (*v*/*v*) of Pickering emulsion, there was already an increase in the contact angle, which indicates a reduction in the hydrophilic character of the films. Figure 5 compares the contact of the drop with the more hydrophilic surface (Control) to the more hydrophobic surface (7.5% *v*/*v*).

Figure 5 shows how the angle θ increases when touching a more hydrophobic surface, indicating that there are fewer interactions with water. This is a desirable effect, especially in applications involving food packaging, since, depending on the storage conditions, a coating that offers resistance to water absorption can be an ally for food preservation [32].

### 3.5. Properties of Starch Films

The results presented in Table 3 show a significant reduction in the water vapor permeability (WVP) of the starch films with the incorporation of Pickering emulsions, particularly at concentrations of 1.0% and 5.0% (*v*/*v*) (0.018 ± 0.01 g·mm/h·m^2^·kPa and 0.016 ± 0.01 g·mm/h·m^2^·kPa, respectively), compared to the control (0.085 ± 0.04 g·mm/h·m^2^·kPa). This suggests that the emulsions effectively improve the water vapor barrier properties, a highly desirable feature for packaging applications where moisture retention is critical.

According to Niro et al. (2021) [13], the enhancement of water vapor barrier properties in films containing emulsions is influenced by the structural interaction between the matrix and the hydrophobic component, particularly the size and dispersion of oil droplets within the matrix. The significant reduction in WVP observed in this study may be attributed to the efficient incorporation of small oil droplets into the starch matrix, which likely formed a more tortuous path for water vapor molecules, limiting their transmission through the film [33].

The preparation method of the Pickering emulsions in this study—utilizing sunflower oil as the oil phase (10%) and BCNF (bacterial cellulose nanofibers) as the stabilizing agent in the aqueous phase (90%)—is critical to the resulting film properties. The use of a high shear force mixer (Ultra Turrax T25) at 10,000 rpm for 10 min enabled the formation of well-dispersed oil droplets within the aqueous phase. This high-energy process is known to create smaller, more stable droplets, which aligns with the observations of Niro et al. (2021) [13], where high-energy methods are emphasized for their effectiveness in achieving better droplet dispersion.

Moreover, the casting technique used for film preparation ensured uniform distribution of the emulsion in the starch matrix. The slow heating of starch to 85 °C followed by the incorporation of glycerol and emulsion at 60 °C likely promoted good miscibility between the starch and emulsion components. The degassing step further prevented the formation of air bubbles, contributing to a more homogenous film structure, which is crucial for achieving the improved barrier properties observed.

The contact angle significantly increased in all films with emulsion compared to the control (49.7 ± 1.5°), reaching up to 71.0 ± 1.4° at a 7.5% (*v*/*v*) concentration. This increase indicates a reduction in the hydrophilic nature of the films, making them more hydrophobic, which is beneficial for moisture resistance.

The tensile strength of the films showed considerable variation with the addition of the emulsion. The control exhibited the highest value (15.2 ± 0.8 MPa), while the 1.0% (*v*/*v*) emulsion concentration reduced this strength to 9.97 ± 0.45 MPa. As mentioned, usually, the addition of lipids and other compounds modifies the mechanical properties of films, mainly reducing the values of tensile strength [13]. However, the film with 2.5% (*v*/*v*) emulsion nearly maintained the original strength (14.2 ± 2.0 MPa), suggesting that this concentration might be optimal for preserving mechanical strength while enhancing other properties.

There was a significant decrease in elongation at break at higher emulsion concentrations. The control showed an elongation of 23.3 ± 2.4%, while the film with 7.5% (*v/v*) emulsion dropped to 4.83 ± 1.1%, indicating that higher emulsion concentrations may make the film stiffer and less flexible. Young’s modulus varied with the addition of the emulsion, with the control showing 589 ± 32 MPa. Although the 1.0% concentration reduced the modulus to 346 ± 16 MPa, the 7.5% (*v*/*v*) concentration significantly increased it to 648 ± 79 MPa, suggesting that films with higher emulsion concentrations are more rigid [34].

In comparison to other starch-based films enhanced with Pickering emulsions reported in the literature, particularly those incorporating essential oils and different forms of cellulose for stabilization, the mechanical properties and WVP values of the films remain superior. This highlights the significant role cellulose nanofibrils play in reinforcing the structure, as evidenced by the notable improvement in tensile strength. Additionally, the inclusion of sunflower oil did not adversely impact the WVP values, as some other oils tend to do. Instead, it contributed to the creation of a more hydrophobic film, resulting in enhanced barrier properties. These characteristics are highly beneficial for food packaging applications, where moisture resistance is a key requirement [34].

## 4. Conclusions

This study demonstrated the significant impact of different concentrations of Pickering emulsions on the mechanical and barrier properties of starch films. The incorporation of the emulsions effectively reduced water vapor permeability (WVP) and increased contact angles, confirming their potential to diminish the hydrophilic nature of the films. Notably, even a low concentration of 1.0% *v*/*v* of the emulsion resulted in a considerable improvement in WVP, while all tested formulations increased the contact angle, indicating enhanced hydrophobicity.

Through tensile testing, the 2.5% concentration emerged as the ideal balance, maintaining mechanical strength close to that of the control film while further enhancing barrier properties and hydrophobicity. This suggests that the 2.5% concentration of Pickering emulsion is optimal for achieving the desired technological properties in starch films for food packaging. Overall, the findings highlight the great potential of Pickering emulsions in advancing the functionality of starch-based films, particularly for applications in sustainable packaging.

## Figures and Tables

**Table 1:** Composition of film-forming suspensions.

Sample	H_2_O (mL)	Emulsion		Starch (g)	Glycerol (g)
		Volume (mL)	BCNF (g)		
Control	50.00	-	-	2.50	0.625
1.0%	49.50	0.50	0.0050	2.50	0.625
2.5%	48.75	1.25	0.0125	2.50	0.625
5.0%	47.50	2.50	0.0250	2.50	0.625
7.5%	46.25	3.75	0.0375	2.50	0.625

**Table 2:** The intensity of the peaks normalized using the starch peak (995 cm^−1^) as a reference.

Bands (cm^−1^)	Control	1.0%	2.5%	5.0%	7.5%
1600	---	0.07	0.01	0.05	0.03
1735	---	0.05	0.10	0.16	0.28
2932	0.11	0.09	0.08	0.20	0.30
3400	0.28	0.29	0.23	0.28	0.24

**Table 3:** Properties of starch films with variation in emulsion concentration.

Properties	Emulsion Concentration (% *v*/*v*)				
	Control	1.0	2.5	5.0	7.5
WVP (g∙mm/h∙m^2^∙kPa)	0.085 ± 0.04 ^a^	0.018 ± 0.01 ^c^	0.046 ± 0.02 ^b^	0.016 ± 0.01 ^c^	0.066 ± 0.03 ^b^
Contact angle (°)	49.7 ± 1.5 ^b^	66.7 ± 1.9 ^a^	64.5 ± 3.1 ^a^	70.0 ± 3.9 ^a^	71.0 ± 1.4 ^a^
Tensile Strength (MPa)	15.2 ± 0.8 ^a^	9.97 ± 0.45 ^b^	14.2 ± 2.0 ^ab^	10.9 ± 0.9 ^ab^	14.8 ± 1.5 ^ab^
Elongation at Break (%)	23.3 ± 2.4 ^a^	22.2 ± 1.6 ^a^	20.9 ± 3.3 ^a^	10.2 ± 0.6 ^b^	4.83 ± 1.1 ^b^
Young’s Modulus (MPa)	589 ± 32 ^ab^	346 ± 16 ^a^	522 ± 87 ^ab^	431 ± 38 ^ab^	648 ± 79 ^b^
Thickness (µm)	54.8 ± 5.3 ^a^	46.4 ± 2.2 ^a^	33.6 ± 3.2 ^b^	43.4 ± 1.0 ^ab^	38.0 ± 1.0 ^b^

The means of the same letter do not differ from each other according to Tukey’s Test, at the 5% significance level.

## Data Availability

Data are contained within the article.
